# Dopamine Increases a Value-Independent Gambling Propensity

**DOI:** 10.1038/npp.2016.68

**Published:** 2016-06-01

**Authors:** Francesco Rigoli, Robb B Rutledge, Benjamin Chew, Olga T Ousdal, Peter Dayan, Raymond J Dolan

**Affiliations:** 1 The Wellcome Trust Centre for Neuroimaging, UCL, London, UK; 2 Max Planck UCL Centre for Computational Psychiatry and Ageing Research, London, UK; 3 Department of Radiology, Haukeland University Hospital, Bergen, Norway; 4 Gatsby Computational Neuroscience Unit, UCL, London, UK

## Abstract

Although the impact of dopamine on reward learning is well documented, its influence on other aspects of behavior remains the subject of much ongoing work. Dopaminergic drugs are known to increase risk-taking behavior, but the underlying mechanisms for this effect are not clear. We probed dopamine's role by examining the effect of its precursor L-DOPA on the choices of healthy human participants in an experimental paradigm that allowed particular components of risk to be distinguished. We show that choice behavior depended on a baseline (ie, value-independent) gambling propensity, a gambling preference scaling with the amount/variance, and a value normalization factor. Boosting dopamine levels specifically increased just the value-independent baseline gambling propensity, leaving the other components unaffected. Our results indicate that the influence of dopamine on choice behavior involves a specific modulation of the attractiveness of risky options—a finding with implications for understanding a range of reward-related psychopathologies including addiction.

## INTRODUCTION

Dopamine has a fundamental role in adaptive behavior, having a well-established influence on reward learning. Phasic dopaminergic bursts encode a prediction error signal (Schultz *et al*, 1997; O'Doherty *et al*, 2003; Tobler *et al*, 2005; D'Ardenne *et al*, 2008; Hart *et al*, 2014) that is a central element in learning rewarding contingencies (Montague *et al*, 1996). Other evidence indicates that dopamine impacts ongoing behavior in a way that is orthogonal to learning, something that speaks to a broader range of functions (Berridge, 2007; Zhang *et al*, 2009) that are subject of much recent work (Palmiter, 2008; Rigoli *et al*, 2016a; Sharot, *et al*, 2009a; Pine *et al*, 2010; Jocham *et al*, 2011; Guitart-Masip *et al*, 2012; Shiner *et al*, 2012; Wunderlich *et al*, 2012; Norbury *et al*, 2013).

One important influence of dopamine relates to risk-taking behavior. Data on rodents and humans, including Parkinson's disease patients, indicate that dopaminergic drugs increase risk-taking behavior (Molina *et al*, 2000; Cools *et al*, 2003; Cools, 2006; St Onge and Floresco, 2008; Rutledge *et al*, 2015). However, the precise mechanisms underlying this effect are unclear. For example, dopamine might affect a baseline (ie, value-independent) propensity towards risk (Kakade and Dayan, 2002; Friston *et al*, 2013; Rutledge *et al*, 2015; Rigoli *et al*, 2016b) or the attribution of subjective value to different reward magnitudes (Berridge, 2007; Zhang *et al*, 2009). Another possibility is that dopamine might encode choice precision, leading to the prediction that boosting dopamine levels would result in increased choice consistency when similar choices are repeatedly presented (Friston *et al*, 2013). Finally, dopamine might affect a normalization process that adapts values to a contextual reward distribution (Tobler *et al*, 2005; Niv *et al*, 2007; Rigoli *et al*, 2016b).

We tested predictions arising out of these varying accounts for the role of dopamine in decision-making in a double-blind, within-subjects design involving administration of either the dopamine precursor levodopa (L-DOPA) or placebo across different sessions. We analyzed participants' choice behavior in a gambling paradigm (Rigoli *et al*, 2016b) where, crucially, several components of risk preference could be disentangled: a baseline risk propensity, a risk propensity dependent on amount/variance, a normalization factor, and an index of choice precision.

## MATERIALS AND METHODS

### Participants

Participants were pre-screened to ensure no existing medical conditions or allergies. Thirty-seven healthy right-handed adults with a maximum weight of 70 kg started the experiment. The weight cutoff was implemented to maximize a putative effect of L-DOPA, given the well-established relationship between weight and the effect of this drug on cognition when the dose is fixed (Zappia *et al*, 2002; Knecht *et al*, 2004; Chowdhury *et al*, 2013; Rutledge *et al*, 2015). None of the participants had any history of head injury, a diagnosis of any neurological or psychiatric condition (including gambling-related or addiction-related pathologies), or was currently on a medication known to affect the central nervous system. Three subjects were excluded from analyses owing to withdrawal after the first session, one participant because she/he felt unwell during the L-DOPA session, and another participant because of an exclusive selection of the option on the left side of the screen (see below). Therefore, the final experimental sample included thirty-two participants (16 females; age: mean=23.4, median=22; SD=4.7; weight: mean=61 kg, median=60 kg, SD=7.3). The study was approved by the University College of London Research Ethics Committee.

### Drug Manipulation and Procedure

Participants were tested at the Wellcome Trust Centre for Neuroimaging, London, in a double-blind, counterbalanced, and placebo-controlled repeated-measures setting consisting of three sessions taking place across different days. Sessions were spaced 3 to 11 days apart and, for each participant, started approximately at the same time to minimize circadian effects.

A first session solely involved performing a gambling task. During the second and third sessions, participants were administered either L-DOPA (150 mg L-DOPA/37.5 mg benserazide; Madopar) or placebo (500 mg ascorbic acid). Drug/placebo administration order was counterbalanced with 14 individuals receiving L-DOPA on the second session. Following drug/placebo administration and a 45-min break, participants performed the same task as in the first session. A subjective state questionnaire was filled three times, namely before drug/placebo administration and before and after the task. Comparison between the different scores did not show statistically significant differences when correcting for multiple comparisons (Supplementary Table S1). Drug/placebo was mixed with orange juice by a researcher who did not conduct testing, assuring a double-blind procedure.

### Experimental Paradigm

**Figure 1:**
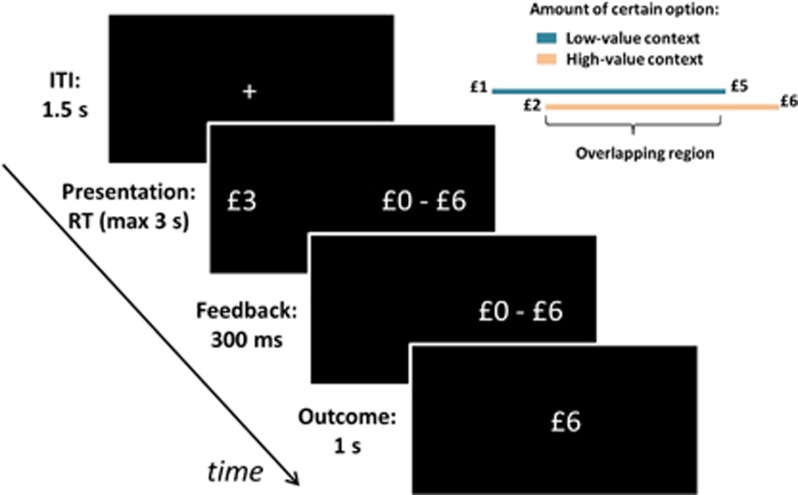
Experimental paradigm. Participants repeatedly made choices between sure gains and gambles associated with either double the sure gain or zero, each with a 50% probability. After choice, the unchosen option disappeared, and after 300 ms, the trial outcome was shown for 1 s. The intertrial interval (ITI) was 1.5 s. Participants performed the task in three separate sessions and in the second and third sessions received either L-DOPA or placebo (ascorbic acid). One outcome was randomly selected from those collected in each session, selected outcomes were added and the resulting amount was paid out to participants. Information on the outcomes selected was provided only after the final session.

In each session, participants performed a 40-min gambling task (Figure 1) where on each trial they chose between a sure monetary amount, which changed trial-to-trial, and an equal probability (50–50) gamble between zero and double the amount. Note that the two options had always equal expected value (EV), corresponding to the sum of monetary amounts associated with possible outcomes, each multiplied by its probability. Crucially, the task was arranged in blocks involving a low-value context, in which EVs were drawn from a £1–£5 uniform distribution, and a high-value context, in which EVs were drawn from a £2–£6 uniform distribution. Participants completed 2 blocks per context, with each block comprising 140 trials and lasting about 10 min. The two contexts alternated, and the initial context was counterbalanced across subjects.

At the beginning of each block, the range of the upcoming reward distribution was displayed for 8 s. For each trial, after a 1.5-s inter-trial interval, the certain monetary amount and gamble were displayed on the left and right sides of the screen, with their positions pseudorandomized. Participants chose the left or right option by pressing the corresponding button on a keypad. Immediately after choice, the chosen option was displayed for 300 ms followed by the outcome for 1 s. Participants had 3 s to respond, and late responses were met with a statement ‘too late' and a zero outcome. On average, participants missed two trials in the first session, and one trial in the second and third sessions. At the end of each session, one outcome was randomly selected and added to an initial participation payment of £30.

For each session, before the task, participants were fully instructed on task contingencies and on the rules determining the participation payment. The monetary amounts selected for each session as additional payment were revealed only after the final session, when payment was carried out.

### Computational Modeling

We characterized choice behavior by implementing the same computational model as in our previous study (Rigoli *et al*, 2016b). The model takes the form of a standard mean-variance return account for which the subjective value of an option *x* is *V*(*x*)=mean(*x*+α variance(*x*. This model has three free parameters: *τ* is a context parameter, which implements a (subtractive) normalization; *α* is a value function parameter, which determines whether reward variance increases (*α*>0) or decreases (*α*<0) the subjective value of risky options; and *μ* is a gambling bias parameter, which determines a baseline propensity to gamble regardless of which options are presented. Taking *A* as the sure monetary amount and *χ* as an indicator of the low-value (*χ*=0) or high-value context ((*χ*=1), then the subjective value of the certain option is *V*_CERT_=*A*−*χτ* so that a positive context parameter *τ* reduces this value in the high-value context. The subjective value of the risky gamble is *V*_GAMB_=*A*−*χτ*+α (*A*−*χτ*)^2^+*μ*. The probability of choosing the gamble is given by a sigmoidal choice rule σ(*V*_GAMB_−*V*_CERT_)=1/(1+(−(*V*_GAMB_−*V*_CERT_)).

The effects postulated by the model in determining gambling probability as a function of different trial EV and different parameter sets are represented in Supplementary Figure S1. This shows that (i) for positive and negative value function parameter *α*, the propensity to gamble for larger EVs increases and decreases, respectively, (ii) larger gambling bias parameter *μ* increases the overall propensity to gamble, (iii) the context parameter *τ* determines whether, in the high compared with low value context, the subjective values attributed to EV increases (*τ*<0) or—as predicted by a value normalization hypothesis—decreases (*τ*>0), impacting on gambling propensity.

We estimated the values of the parameters using function *fminsearch* in Matlab, without enforcing any constraint. We took the best fitting out of 50 locally optimal parameter sets, sampling initial values randomly from Gaussian distributions with mean 0 and SD 2. For estimation, trials with RTs longer than 3 s (ie, associated with ‘too late' statement) were discarded (implying that 53 615 trials remained for all subjects and sessions). We used Bayesian model comparison to compare our model with simpler versions in which one or two parameters were fixed, and with more complex versions in which one or more parameters were each replaced by separate parameters for each of the three sessions. Bayesian Information Criterion (BIC) scores were computed for each model fit and summed across subjects. BIC scores penalize for parameter number and the model with the lowest BIC score is preferred.

## RESULTS

### Effects of Time

We first sought to replicate previous findings from a study using the identical task (Rigoli *et al*, 2016b). As before, we found that the average gambling percentage did not differ from 50% (session 1 (S1): Mean=45.54, SD=19.99, t(31)=1.26, *p*=0.22; session 2 (S2): Mean=45.93, SD=22.49, t(31)=1.0, *p*=0.31; session 3 (S3): Mean=47.40, SD=23.23, t(31)=0.6, *p*=0.53; two-tailed *p*=0.05 is used as significant threshold throughout). We estimated a logistic regression model of choice including the trial EV as predictor (remember that, by design, the two options always had equivalent EV). The EV-related beta weight (a measure we refer to as gambling slope) did not differ significantly from zero, indicating that participants did not gamble more with large or small EVs (S1: t(31)=0.2, *p*=0.84; S2: t(31)=0.3, *p*=0.80; S3: t(31)=0.3, *p*=0.77). We found no difference in gambling percentage between low- and high-value contexts (S1: t(31)=−0.306, *p*=0.762; S2: t(31)=−0.891, *p*=0.380; S3: t(31)=−0.968, *p*=0.341) even when considering gambles for EVs that overlapped in the two contexts (S1: t(31)=0.127, *p*=0.900; S2: t(31)=−0.574, *p*=0.570; S3: t(31)=−0.403, *p*=0.689).

**Figure 2:**
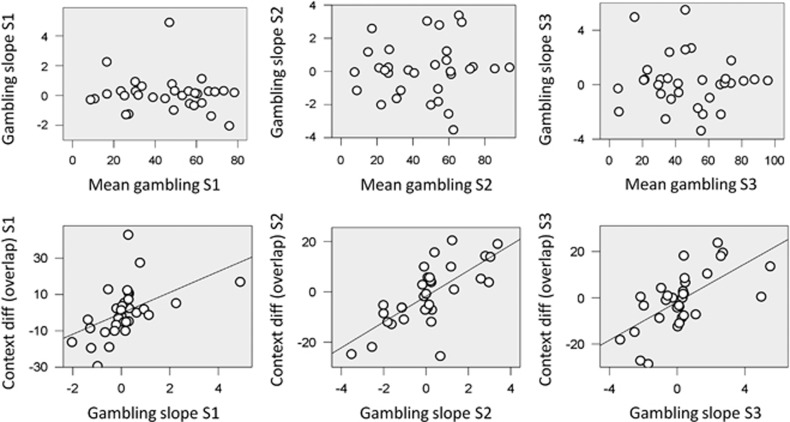
Relationship among decision indexes within each session. The first row shows the relationship between mean gambling percentage and gambling slope, corresponding to the beta weight associated with EV in a logistic regression model of gambling choice. The second row reports the relationship between gambling slope and the difference in gambling percentage between overlapping EVs of the two contexts (£2–£5 range for low- minus high-value context). Different columns indicate sessions (S1=session 1; S2=session 2; S3=session 3). For the first relationship, in no case did we observe a significant correlation (*p*>0.5), whereas for the second relationship, we observed a significant correlation in all sessions (*p*<0.005).

There was a correlation between the intercept of the logistic regression and gambling slope (S1: r(32)=−0.974, *p*<0.001; S2: r(32)=−0.962, *p*<0.001; S3: r(32)=−0.976, *p*<0.001; note that estimation of the latter is unaffected by which intercept is assumed), whereas there was no correlation between average gambling percentage and gambling slope (Figure 2; Supplementary Figure S1: r(32)=−0.121, *p*=0.508; S2: r(32)=0.063, *p*=0.732; S3: r(32)=−0.077, *p*=0.676). This suggests that average gambling percentage and tendency to gamble with large or small EV (captured by the gambling slope) are two different dimensions.

Value normalization predicts that participants with a gambling preference for larger EVs gamble more when EVs that overlap across contexts (ie, in the £2–£5 range) are relatively larger, namely in the low-value context. Conversely, participants with a gambling preference for smaller EVs would gamble more when overlapping EVs are relatively smaller, namely in the high-value context. Therefore, we predicted that the individual gambling slope correlated with the difference in gambling for overlapping EVs across contexts. This was confirmed in all sessions by partial correlations between gambling slope and gambling percentage in the low-value context controlling for the high-value context (S1: r(29)=0.454, *p*=0.010; S2: r(29)=0.511, *p*=0.003; S3: r(29)=0.484, *p*=0.006). Altogether, these data replicate previous findings (Rigoli *et al*, 2016b). The findings indicate that two partially independent factors contribute to risk-taking behavior, namely a baseline risk propensity (captured by the average gambling) and a preference dependent on EV (captured by the gambling slope, which was uncorrelated with the average gambling). Consistent with value normalization, participants who prefer gambling with large/small EVs also gamble more when equivalent EVs are relatively larger/smaller compared with the context (as captured by the correlation between gambling slope and gambling difference for overlapping EVs).

Next, we investigated cross-section stability in choice behavior. We did not observe any systematic difference in gambling percentage across sessions (F(2,62)=0.4, *p*=0.671) nor in the difference in gambling percentage between contexts (for all choices: F(2,62)=0.330, *p*=0.720; for overlapping EVs: F(2,62)=0.304, *p*=0.739). Also, we observed a correlation across sessions (Supplementary Figure S2) for the gambling percentage (S1 and S2: r(32)=0.861, *p*<0.001; S1 and S3: r(32)=0.834, *p*<0.001; S2 and S3: r(32)=0.843, *p*<0.001), the gambling slope (S1 and S2: r(32)=0.726, *p*<0.001; S1 and S3: r(32)=0.833, *p*<0.001; S2 and S3: r(32)=0.849, *p*<0.001), and the contextual differences for overlapping EVs (S1 and S2: r(32)=0.505, *p*=0.003; S1 and S3: r(32)=0.594, *p*<0.001; S2 and S3: r(32)=0.682, *p*<0.001), suggesting these measures underpin stable traits.

To investigate whether dopamine is linked with choice precision (Friston *et al*, 2013), we estimated two components of this putative factor. The first is the absolute value of the gambling slope, indicating how much the EV is taken into consideration during choice. Note that a larger absolute gambling slope entails an increased choice precision because of greater choice consistency for similar EVs. The second is the absolute difference between average gambling percentage and 50%, a measure we refer to as baseline choice precision, as it indicates overall choice consistency. Both measures were correlated across sessions (absolute gambling slope: S1 and S2: r(32)=0.618, *p*<0.001; S1 and S3: r(32)=0.760, *p*<0.001; S2 and S3: r(32)=0.707, *p*<0.001; baseline choice precision: S1 and S2: r(32)=0.688, *p*<0.001; S1 and S3: r(32)=0.643, *p*<0.001; S2 and S3: r(32)=0.743, *p*<0.001). The absolute gambling slope changed across sessions (F(2,62)=7.249, *p*=0.001), an effect driven by a smaller absolute gambling slope in the first session (S1 *vs* S2: t(31)=−2.95, *p*=0.006; S1 *vs* S3: t(31)=−3.722, *p*=0.001; S2 *vs* S3: t(31)=−0.681, *p*=0.501). An increased absolute gambling slope after the first session indicates that choice dependency on EV is enhanced after a first exposure to the task. This may be connected to evidence showing a tendency to rely more on a strategy after this strategy is repeatedly selected (Sharot *et al*, 2009b). Baseline choice precision did not differ significantly across sessions (F(2,62)=1.084, *p*=0.345).

### Model-Based Analysis

According to model comparison, in the best model of choice behavior (see Materials and Methods), the gambling bias parameter *μ* and the value function parameter *α* differed across sessions while the context parameter *τ* did not (Table 1; Supplementary Figure S3). To assess whether the best model was better than chance for all subjects, we compared individual BIC scores of the best model with BIC scores of the random model. For every single participant, the BIC score of the best model was smaller than the BIC score of the random model. Thus, no participant was better fit by pure chance.

The parameters in the model are expected to provide quantitative characterizations of elements of behavior. As predicted, the context parameter *τ* was significantly positive (t(31)=3.03, *p*=0.005), consistent with value normalization in which subjective values were rescaled to the contextual reward distribution. Note that in the model, *V*_GAMB_−*V*_CERT_=α (*A*−*χτ*)^2^+*μ*. This is similar to a simple logistic regression, where the value function parameter *α* corresponds to a slope parameter and the gambling bias parameter *μ* corresponds to an intercept. According to this, the value function parameter *α* was correlated with the gambling slope, suggesting that both capture a preference to gamble with large or small trial amount (S1: r(32)=0.990, *p*<0.001; S2: r(32)=0.985, *p*<0.001; S3: r(32)=0.957, *p*<0.001), but it was not correlated with the average gambling percentage (S1: r(32)=−0.062, *p*=0.735; S2: r(32)=0.133, *p*=0.470; S3: r(32)=0.019, *p*=0.917). The gambling bias parameter *μ* correlated with the intercept of the logistic regression model of choice having the EV as predictor (S1: r(32)=0.981, *p*<0.001; S2: r(32)=0.976, *p*<0.001; S3: r(32)=0.941, *p*<0.001). Similarly to the logistic regression, the gambling bias parameter *μ* and the value function parameter *α* were inversely correlated (S1: r(32)=−0.892, *p*<0.001; S2: r(32)=−0.879, *p*<0.001; S3: r(32)=−0.871, *p*<0.001). However, note that a correlation between intercept and slope does not affect the estimate of the slope and maintains the two parameters identifiable during model fitting (see below for recovery analyses).

To analyze whether the model is able to explain raw behavioral analyses, we used the model together with subject-specific parameters estimates to generate simulated data (from the same number of trials as in the real experiment; ie, 560 trials per subject) and performed exactly the same behavioral analyses on the simulated data that we had used for the experimental data. For this analysis, we focused on the first session alone and compared a model with the three parameters *α*, *μ,* and *τ* against models where one of these parameters was fixed to zero. Consistent with real data, the full model replicated the lack of correlation between average gambling and the gambling slope (ie, the slope of the logistic regression having EV as regressor; r(32)=−0.091, *p*=0.624), while a correlation emerged when data were simulated using a model without the gambling bias parameter *μ* (r(32)=0.78, *p*<0.001). Moreover, again consistent with empirical data, the full model replicated the partial correlation between the gambling slope and the gambling for choices that overlapped across context in the low-value context controlling for the high value-context (r(29)=0.490, *p*=0.007). This was not obtained when data were simulated using a model without the value function parameter *α* (r(32)=0.03, *p*=0.89) or without the context parameter *τ* (r(32)=−0.08, *p*=0.663). We also inspected that the model which best explained the data (ie, with parameter *α* and *μ* varying and *τ* not varying across sessions) could replicate the raw behavioral analyses. Hence, we simulated data with this model and found a lack of correlation between average gambling and the gambling slope (S1: r(32)=−0.05, *p*=0.786; S2: r(32)=−0.02, *p*=0.913; S3: r(32)=−0.09, *p*=0.624), and a partial correlation between the gambling slope and the gambling for choices that overlapped across context in the low-value context controlling for the high value-context (S1: r(29)=0.40, *p*=0.032; S2: r(29)=0.52, *p*=0.003; S3: r(29)=0.53, *p*=0.003). Next, we fitted the model to the simulated data and the estimated parameters were correlated with the true parameters across individuals (*τ*: r(29)=0.85, *p*<0.001; S1: *α*: r(29)=0.97, *p*<0.001; *μ*: r(29)=0.92, *p*<0.001; S2: *α*: r(29)=0.98, *p*<0.001; *μ*: r(29)=0.91, *p*<0.001; S3: *α*: r(29)=0.95, *p*<0.001; *μ*: r(29)=0.94, *p*=0.001). Collectively, these analyses validate the mean-return model and show that it is able to reproduce raw behavioral analyses and that parameters can be estimated reliably.

Next, we assessed cross-session stability of parameters. The individual gambling bias parameters *μ* were correlated across sessions (S1 and S2: r(32)=0.797, *p*<0.001; S1 and S3: r(32)=0.854, *p*<0.001; S2 and S3: r(32)=0.882, *p*<0.001) as well as the value function coefficients *α* (S1 and S2: r(32)=0.667, *p*<0.001; S1 and S3: r(32)=0.775, *p*<0.001; S2 and S3: r(32)=0.813, *p*<0.001), consistent with these measures underlying stable individual risk attitudes.

Recent evidence from rodent data suggests that dopamine mediates the effect of recent reward history on risky choice (Stopper *et al*, 2014). We asked whether participants were affected by previous outcomes by estimating, for each session, a logistic regression model of choice including reward prediction error at the previous trial (ie, equal to zero for choices of the sure option, equal to the monetary outcome minus the EV for choices of gamble) as predictor. Computing the BIC for this model and for a model including an intercept parameter only, and summing BICs for all sessions, the simpler model was preferred (BIC=63798 *vs* BIC=63491), suggesting that participants' choices were not affected by previous outcomes.

### Effects of Drug Manipulation

Our main research goal was to investigate effects of L-DOPA compared with placebo on risk-taking behavior. On the basis of a hypothesis that dopamine boosts overall risk-taking, an increased average gambling percentage is predicted under L-DOPA (Kakade and Dayan, 2002; Friston *et al*, 2013; Rutledge *et al*, 2015; Rigoli *et al*, 2016b). By contrast, based on the idea that dopamine influences the value assigned to different reward magnitudes, a difference in gambling slope would be predicted across drug/placebo (Berridge, 2007; Zhang *et al*, 2009). Contrary to most previous studies, these two predictions could be disentangled as the two indexes were uncorrelated in the task. Furthermore, we were able to consider the possibility that L-DOPA influences choice precision (Friston *et al*, 2013), operationalized as baseline choice precision and absolute gambling slope. Finally, we considered the impact of L-DOPA on contextual normalization by computing, for each subject, the product between the gambling slope and the difference in gambling percentage for overlapping EVs across contexts. We refer to this measure as corrected context effect and it was implemented to capture a propensity for subjects who preferred to gamble with large/small EVs also to gamble more when the same EVs were relatively larger/smaller. Consistent with contextual normalization, the corrected context effect was significantly positive in all sessions (S1: t(31)=2.7, *p*=0.011; S2: t(31)=3.45, *p*=0.002; S3: t(31)=3.39, *p*=0.002). This index was expected to be affected by our drug manipulation according to a hypothesis that different levels of dopamine alter reward contextual normalization. A final prediction we considered is that boosting dopamine levels would imply that all rewards would appear subjectively less or more valuable, resulting in changes of average gambling in participants with convex and concave subjective value functions (but in opposite directions).

**Figure 3:**
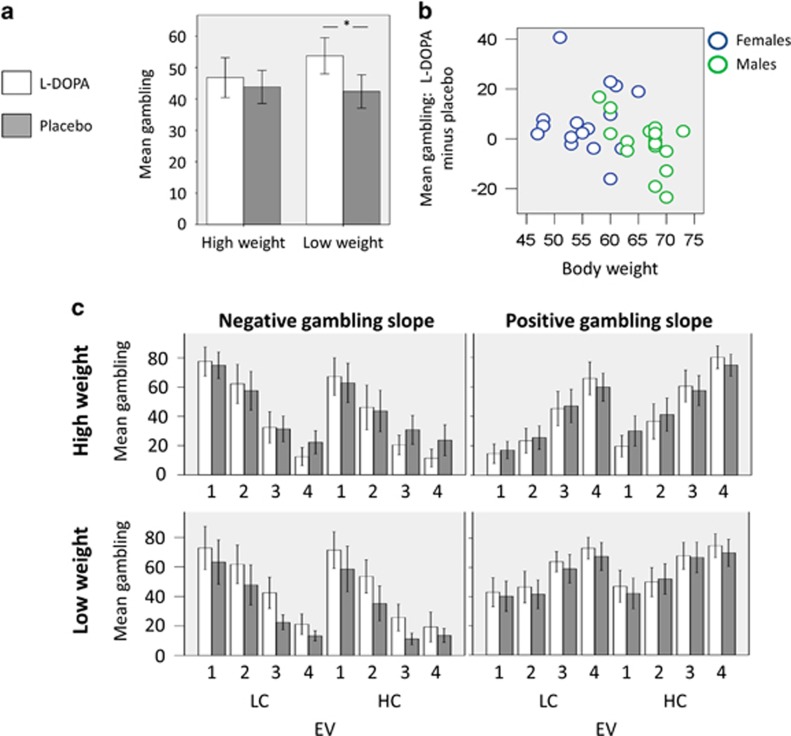
(a) Mean gambling percentage as a function of drug (L-DOPA *vs* placebo) and body weight (high *vs* low body weight). Larger average gambling percentage under L-DOPA compared with placebo was observed in low-weight (t(15)=2.2, *p*=0.044) but not high-weight participants (t(15)=0.5, *p*=0.625). (b) Relationship between the effect of L-DOPA (relative to placebo) on average gambling percentage and body weight, where blue circles represent females (*n*=16) and green circles represent males (*n*=16). We observed a significant inverse partial correlation between average gambling percentage under L-DOPA and body weight, controlling for average gambling percentage under placebo (b; r(29)=−0.378, *p*=0.043). A significant inverse partial correlation was found within males (r(13)=−0.516, *p*=0.049) but not within females (r(13)=−0.078, *p*=0.782), possibly because of a ceiling effect, as females had substantial lower weight than males. This suggests that weight and not gender mediated an effect of drug on average gambling percentage. (c) Difference in gambling percentage between L-DOPA (white bars) and placebo (grey bars) in different groups of subjects and different conditions. Participants are grouped according to two dimensions: (i) weight, where data for high and low body weight groups, created based on a median split, are, respectively, displayed in the first and second row of panels; (ii) gambling slope (ie, the beta weight associated with EV in a logistic regression model of gambling choice), where data for participants with negative and positive gambling slope in the first session are displayed in the first and second column of panels, respectively. For each group of participants, mean gambling percentage (on the *y* axis) is reported for each of four standardized bins of increasing EV separated for each context (LC: low-value context; HC: high-value context). Considering low body weight participants alone, this figure shows an increased mean gambling percentage with L-DOPA compared with placebo in all EV bins and contexts and for both positive and negative gambling slope participants (bin 3 in high value context for positive gambling slope participants is an exception but not statistically significant). This is confirmed by a lack of an interaction between the different bins and the drug/placebo manipulation.

Given the same dosage (150 mg) was administered to all participants and given previous reports that the effect of L-DOPA can depend on body weight (Zappia *et al*, 2002; Knecht *et al*, 2004; Chowdhury *et al*, 2013; Rutledge *et al*, 2015), we considered the possibility that weight mediates the effect of L-DOPA. In keeping with previous literature (Zappia *et al*, 2002; Knecht *et al*, 2004; Chowdhury *et al*, 2013; Rutledge *et al*, 2015), in our analyses, we used body weight and not, for instance, the related measure of body mass index. To account for body weight, participants were assigned to low/high-weight groups (based on a median split) and mixed-effect ANOVAs on the behavioral measures were run with drug as within-subjects factor and weight grouping as between-subjects factor. Results of these analyses are reported in Table 2 and show no main or interaction effects except for an interaction effect on average gambling (Figure 3a and c; F(1,30)=4.96, *p*=0.034). More detailed analyses showed that the drug–weight interaction was driven by a larger average gambling percentage under L-DOPA compared with placebo in low-weight (Figure 3a–c; t(15)=2.2, *p*=0.044) but not high-weight participants (Figure 3a and c; t(15)=0.5, *p*=0.625). This was confirmed by a similar analysis where we observed a significant inverse partial correlation between average gambling percentage under L-DOPA and body, controlling for average gambling percentage under placebo (Figure 3b; r(29)=−0.378, *p*=0.043). This supports the idea that L-DOPA boosts risk-taking behavior. The role of weight is not surprising given the well-established link between this variable and effective drug dose.

To assess whether the role of weight as mediator was confounded by age, participants were assigned to young/old groups (based on a median split) and a mixed-effect ANOVA on average gambling percentage was run with drug as within-subjects factor and weight grouping as between-subjects factor, but we found no interaction effect (F(1,30)=0.16, *p*=0.688). However, a similar analysis having gender as between-subjects factor showed a significant interaction effect (F(1,30)=4.54, *p*=0.041), and we observed higher weight in males than females (t(30)=6.21, *p*<0.001). This raises the possibility that gender might explain the weight–drug interaction effect on average gambling percentage. If this was the case, we would expect no relationship between weight and effect of L-DOPA (relative to placebo) on average gambling percentage within subgroups, namely within males and females. Contrary to this prediction, we found a significant inverse partial correlation in males between average gambling percentage under L-DOPA and body weight, controlling for average gambling percentage under placebo (r(13)=−0.516, *p*=0.049). No partial correlation was found in females (r(13)=−0.078, *p*=0.782), possibly owing to a ceiling effect, as females were substantially lighter than males.

In addition, we computed the average gambling percentage for L-DOPA minus placebo and estimated a stepwise regression model of this variable where both weight and gender were considered as predictors. Independent of the specific stepwise method used (ie, stepwise, forward, or backward), the regression model always included weight (t(31)=2.34, *p*=0.026) and excluded gender (t(31)=−0.60, *p*=0.55) as predictor. These analyses are more consistent with the possibility that weight and not gender mediated the effect of drug on average gambling percentage, also in line with previous findings (Rutledge *et al*, 2015).

Contingent on whether participants' gambling slope was positive or negative in the first session, subjects were also assigned to positive/negative gambling slope groups. To test for specific effects of EV, we grouped the different EVs in two bins, one for EVs that are negative relative to the contextual mean EV (£1–3 and £2–4 range in the low- and high-value context, respectively) and the other for EVs that are positive relative to the contextual mean EV (£3–5 and £4–6 range in the low- and high-value context, respectively). We implemented a mixed-effect ANOVA with drug and EV bins as within-subjects factors, and weight and gambling slope grouping as between-subjects factors. We observed a significant drug–weight interaction effect (F(1,28)=4.96, *p*=0.034) plus, unsurprisingly, a gambling slope–EV bin interaction effect (F(1,28)=46.16, *p*<0.001); all other effects were not statistically significant.

Overall, these data suggest that boosting dopamine levels with L-DOPA enhances an average gambling propensity and this effect does not interact with EV or with participants' stable preferences to gamble more with small or large EVs.

## DISCUSSION

Investigating choice under risk is an important probe of the role of dopamine in decision-making. In general, drugs that boost dopamine levels enhance the propensity to take risks (Molina *et al*, 2010; Cools *et al*, 2003; St Onge and Floresco, 2008; Rutledge *et al*, 2015). However, this can be explained by one or more mechanisms. Dopamine might increase the overall attractiveness of gambling without regard to the stake values (Kakade and Dayan, 2002; Friston *et al*, 2013; Rutledge *et al*, 2015; Rigoli *et al*, 2016b), influence the value attributed to different reward magnitudes (Berridge, 2007; Zhang *et al*, 2009), boost choice precision (Friston *et al*, 2013), mediate the influence of past rewards (Frank *et al*, 2004; Pessiglione *et al*, 2006; Stopper *et al*, 2014), or impact on contextual reward normalization (Tobler *et al*, 2005; Rigoli *et al*, 2016b).

We manipulated dopamine availability using the dopamine precursor L-DOPA while subjects performed a task designed to isolate and test the above possibilities. We observed that the overall mean frequency of gambling was uncorrelated with the preference to gamble with large or small EVs. This finding highlights two independent components of risk preference, one tied to a baseline risk propensity and the other to a subjective value function. We also observed reward normalization because a difference in gambling across contexts for equivalent EVs interacted with the preference to gamble with large or small EVs. In other words, participants who preferred gambling with large/small EVs also gambled more when equivalent EVs were larger/smaller relative to the context. As subjects performed experimental sessions over multiple days, this allowed us to show that individual risk preferences were stable across sessions.

Our main finding was that under L-DOPA compared with placebo, low body weight participants (a proxy for higher overall dopamine effect) exhibited increased mean gambling preference. The interaction with body weight was not surprising given the use of a fixed drug dosage and the well-established relationship between body weight and drug effects (Zappia *et al*, 2002; Knecht *et al*, 2004; Chowdhury *et al*, 2013; Rutledge *et al*, 2015). Though body weight was related with gender and hence an impact of the latter variable cannot be completely ruled out, the fact that the effect of drug correlated with weight within males suggests that weight and not gender mediated the effect of L-DOPA.

The dopaminergic effect on a baseline risk propensity did not depend on the values of the various options. A previous study reported a similar effect on a measure akin to the baseline risk propensity implemented here and found an effect on gambling for gains but not for losses (Rutledge *et al*, 2015). However, in that study, it was impossible to tell whether the effect depended on gains themselves or on the fact that gain trials were as a whole preferred to all other trials because the alternative trial types all involved losses. By contrast, the task here only involved gain trials, with small and large potential gains having negative and positive valence relative to the average reward for all trials. We showed that increased gambling under L-DOPA emerges in all gain trials, irrespective of relative preference, because an increased gambling under L-DOPA was evident also for EVs below the average for all trials.

The value-independent (but, according to Rutledge *et al* (2015), potentially valence-dependent) effect of dopamine on risk has been interpreted as a form of Pavlovian approach (Dayan *et al*, 2006; Rigoli *et al*, 2012, 2016c; Rutledge *et al*, 2015). This might arise from an exploration bonus associated with potentially surprising outcomes or, more formally, from a value attributed to outcome entropy linked to constructs such as curiosity, intrinsic motivation, and information gain (Kakade and Dayan, 2002; Tishby and Polani, 2011; Friston *et al*, 2013; Rigoli *et al*, 2016b). Such an explanation would chime with evidence that dopamine, risk preference, and curiosity all decrease with age (Giambra *et al*, 1992; Deakin *et al*, 2004) and with the established relationship between dopamine and the personality trait of sensation-seeking (Ratsma *et al*, 2001; Norbury *et al*, 2013). Another possibility is that boosted dopamine levels act to increase the probability estimate of positive outcomes (Friston *et al*, 2013), here corresponding to the belief that larger outcomes are more likely after gambling. This is also consistent with evidence of an increased optimism bias under L-DOPA (Sharot *et al*, 2009a) and with findings showing that amphetamine (a drug that increases the levels of dopamine and norepinephrine) transforms negative arousal (fear) into positive arousal (excitement) (Knutson *et al*, 2004).

Our data provided no support for the suggestion that boosting dopamine levels influences the convexity or concavity of the value function (Berridge, 2007; Zhang *et al*, 2009). This indicates that augmented risk-taking behavior under L-DOPA cannot be interpreted as an increased weight given to larger reward amounts. L-DOPA also left unchanged the precision of choices (Friston *et al*, 2013), measured either by the distance between the mean gambling percentage and 50% or by the absolute gambling slope (indicating how much monetary amount was taken into account). Further, we recently observed a link between choice and neural adaptation to the contextual reward distribution in the dopaminergic midbrain (Rigoli *et al*, 2016b). However, a prediction that the extent of individual choice contextual adaptation would be modulated by dopamine levels was not supported here.

We also considered an hypothesis that boosting dopamine levels would imply that all rewards would appear subjectively less or more valuable, resulting in changes of average gambling in participants with convex and concave subjective value functions (but in opposite directions). However, our findings also did not support this prediction, suggesting that dopaminergic levels might be unrelated to reward normalization.

That dopamine and dopaminergic manipulations affect learning is well established (Frank *et al*, 2004; Pessiglione *et al*, 2006; Shohamy *et al*, 2006; Rutledge *et al*, 2009; Guitart-Masip *et al*, 2012; Shiner *et al*, 2012; Chowdhury *et al*, 2013), and this has been shown to be relevant in decision-making under risk (Stopper *et al*, 2014). However, the effects observed in our study are unlikely to depend on learning because in our task, we found no evidence that choice was affected by previous outcomes. We stress that our findings do not go against the hypothesis of dopamine being involved in learning, but demonstrate instead that manipulating dopaminergic transmission has effects on behavior that go beyond dopamine's established role in learning. We also highlight some important limitations of the study. First, the monetary amounts used are small, and hence whether this effect is present also with larger amounts that may be more relevant to real-world economic decisions remains an open question. Second, the use of monetary incentives leaves unanswered the question as to whether our effects can be observed also in the presence of other incentives, such as food or drugs including those known to affect the dopamine system. Third, dopaminergic effects on cognition can follow an inverted U-shape function (Cools, 2006), raising the possibility that a gambling propensity might be maximal at a certain dopamine level and decrease as one departs from this level. Using multiple dosages in future research might reveal a non-linear relationship between dopaminergic transmission and risk-taking behavior. Finally, a question that remains open is whether the effect of L-DOPA described here is more closely related to an influence on phasic or tonic dopaminergic activity, as it is still unknown whether L-DOPA acts more on phasic or tonic processes (Floresco *et al*, 2003; Niv *et al*, 2007), a question that techniques like pharmacology combined with microdialysis and cyclic voltammetry could address (Hart *et al*, 2014; Hamid *et al*, 2016).

In conclusion, we studied the role of dopamine in decision-making using a gambling task that allowed us to separate several aspects of choice behavior. Boosting dopamine levels enhanced a baseline propensity to take risks, but had no effect on any other aspect of choice behavior. This raises the possibility that dopamine modulates the attractiveness of surprising outcomes or increases an optimism bias. Such a finding is of potential interest in clinical populations, and suggests the possibility that patients with abnormal dopaminergic functioning or under dopaminergic treatment (eg, Parkinson's disease patients) are differently attracted by surprising outcomes. This may help not only in understanding psychopathologies such as gambling and drug abuse, with well-known links with dopamine and risk behavior, but also other conditions in which dopamine is implicated in connection with aberrant salience (Kapur, 2003), such as schizophrenia and attention deficit hyperactivity. In addition, these and similar data may suggest that one should consider measures associated with endogenous dopamine levels, such as genetic information (Frank *et al*, 2007), as possible biomarkers for screening individuals at risk of pathological gambling.

## FUNDING AND DISCLOSURE

The authors declare no conflict of interest.

## Figures and Tables

**Table 1:** Comparison Among Behavioral Models of Choice Behavior

**Free parameters**	**BIC**	**Negative Log-likelihood**	**Pseudo-R**^**2**^
Random	74326	37163	0
*μ*	64536	32265	0.13
*α*	65099	32546	0.12
*τ*	74270	37132	0
*μ, α*	54441	27214	0.27
*μ, τ*	64774	32181	0.13
*α, τ*	64830	32409	0.13
*μ, α, τ*	53798	26890	0.28
*μ*_1_*, μ*_2_*, μ*_3_*, α, τ*	52769	26369	0.29
*μ, α*_1_*, α*_2_*, α*_3_*, τ*	52298	26133	0.30
*μ, α, τ*_1_*, τ*_2_*, τ*_3_	53081	26525	0.29
*μ*_1_*, μ*_2_*, μ*_3_*, α*_1_*, α*_2_*, α*_3_*, τ*	*50996	25476	0.32
*μ*_1_*, μ*_2_*, μ*_3_*, α, τ*_1_*, τ*_2_*, τ*_3_	52604	26280	0.29
*μ, α*_1_*, α*_2_*, α*_3_*, τ*_1_*, τ*_2_*, τ*_3_	52471	26213	0.29
*μ*_1_, *μ*_2_*, μ*_3_*, α*_1_*, α*_2_*, α*_3_*, τ*_1_*, τ*_2_*, τ*_3_	51148	25546	0.32

Models are estimated from all trials excluding missed trials. The first column reports the free parameters of each model. The baseline model includes a gambling bias parameter *μ*, a value function parameter *α*, and a context parameter *τ*. This model is compared with simpler models where one or more of these parameters are fixed, and with more complex models where one or more of these parameters are replaced by three parameters, one for each session (indicated by subscripts). According to BIC statistic, the best model (marked with an asterisk) includes separate baseline gambling parameters *μ* and value function parameters *α* for each session while the context coefficient parameter *τ* is equivalent across sessions. Negative log-likelihood and Pseudo-R^2^ of models are reported in the third and fourth column, respectively.

**Table 2:** Effects of Drug/Placebo Manipulation on the Different Behavioral Measures

**Behavioral measure**	**Drug: main effect**	**Weight: main effect**	**Drug–weight interaction**
Average gambling percentage	F(1,30)=1.66, *p*=0.208	F(1,30)=0.42, *p*=0.520	F(1,30)=4.96, *p*=0.034*
Gambling slope	F(1,30)=0.00, *p*=0.960	F(1,30)=0.00, *p*=0.943	F(1,30)=1.34, *p*=0.256
Corrected context effect	F(1,30)=1.76, *p*=0.195	F(1,30)=0.27, *p*=0.604	F(1,30)=0.03, *p*=0.857
Absolute gambling slope	F(1,30)=1.41, *p*=0.240	F(1,30)=0.88, *p*=0.357	F(1,30)=0.09, *p*=0.767
Baseline choice precision	F(1,30)=0.54, *p*=0.390	F(1,30)=0.00, *p*=0.948	F(1,30)=2.30, *p*=0.140
Gambling bias parameter μ	F(1,30)=1.00, *p*=0.325	F(1,30)=0.47, *p*=0.830	F(1,30)=1.40, *p*=0.246
Value function parameter *α*	F(1,30)=0.15, *p*=0.698	F(1,30)=0.241, *p*=0.627	F(1,30)=0.756, *p*=0.391

To account for body weight, participants were assigned to low/high-weight groups (based on a median split) and mixed-effect ANOVAs on the behavioral measures were run with drug as within-subjects factor and weight grouping as between-subjects factor. Results of these analyses are reported here. The interaction effect on average gambling percentage alone is significant and is marked with an asterisk.
