# Supplementary material for: Dopamine Increases a Value-Independent Gambling Propensity
Source: Neuropsychopharmacology. 2016 Jun 1;41(11):2658–67. doi: 10.1038/npp.2016.68 (PMC5026733; doi:10.1038/npp.2016.68)
Supplement: Supplementary Information [file npp201668x1.docx]

**Supplemental material**


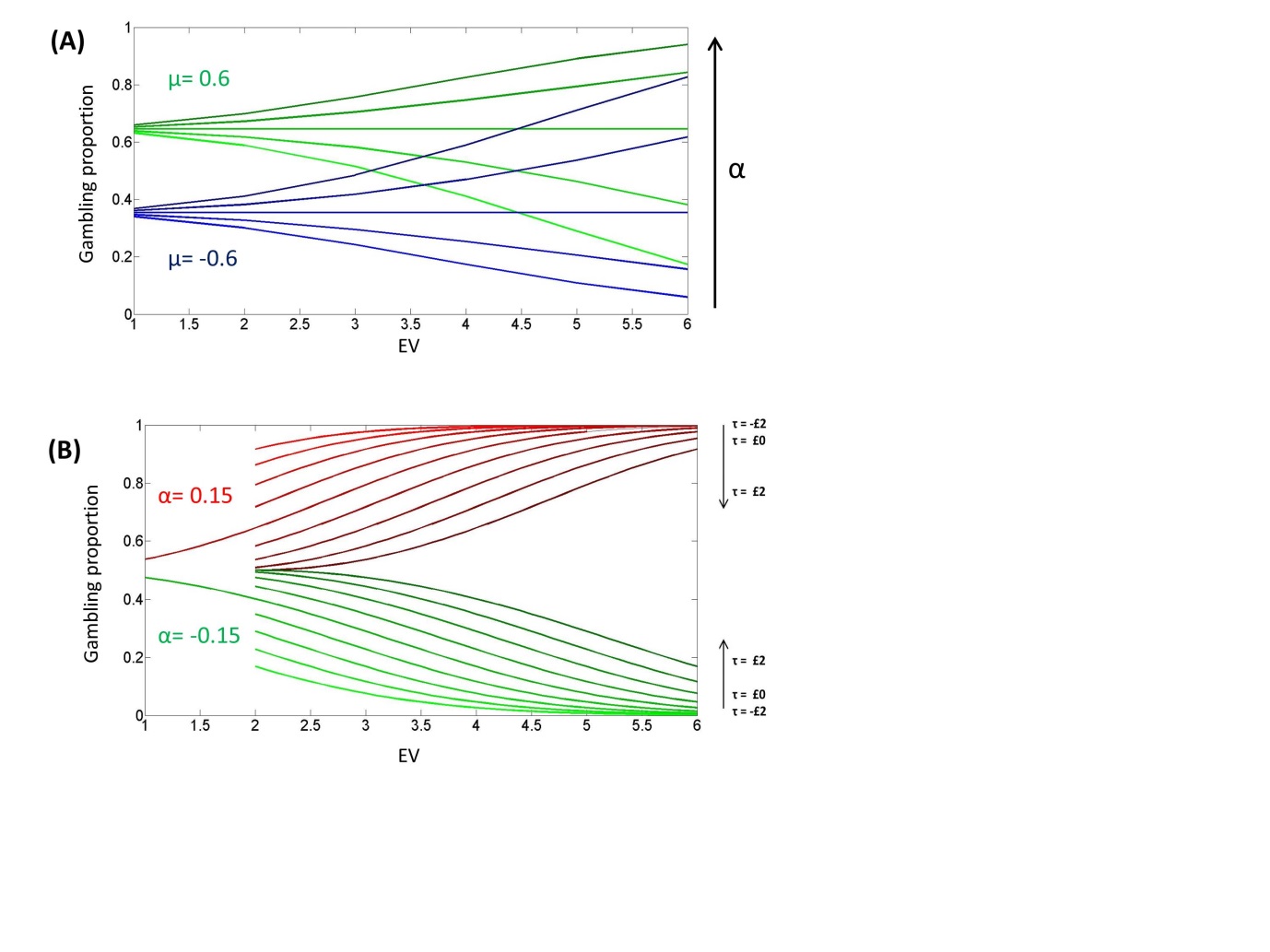


**Figure S1.** Plots of the gambling probability as a function of trial EV (remember that the two options always had equivalent EV) for a set of simulated agents with specific parameters. **A:** Effect of varying the value function parameter α (from $£^{-1}$-0.06 to $£^{-1}$ 0.06 with increases in $£^{-1}$0.03 steps, represented along a bright-to-dark gradient) and the gambling bias parameter μ (green and blue lines implement μ=£0.6 and μ=-£0.6 respectively). It is evident that α determines the tendency to gamble for large or small amounts, while μ is analogous to an intercept parameter reflecting the tendency to gamble for an hypothetical EV of zero. Here the context parameter τ is set to zero **B:** Effect of varying the value function parameter α and the context parameter τ. Red lines represent agents with a positive value function coefficient α (equal to $£^{-1}$0.15) and green lines represent agents with a negative alpha (equal to $£^{-1}$-0.15). Behaviour of agents with τ equal to zero is represented by lines extending from EV=£1 to EV=£6. For the high-value context extending from EV=£2 to EV=£6, agents with different τ are plotted in which τ increases in £0.5 steps from -£2 to £2 along a bright-to-dark gradient.


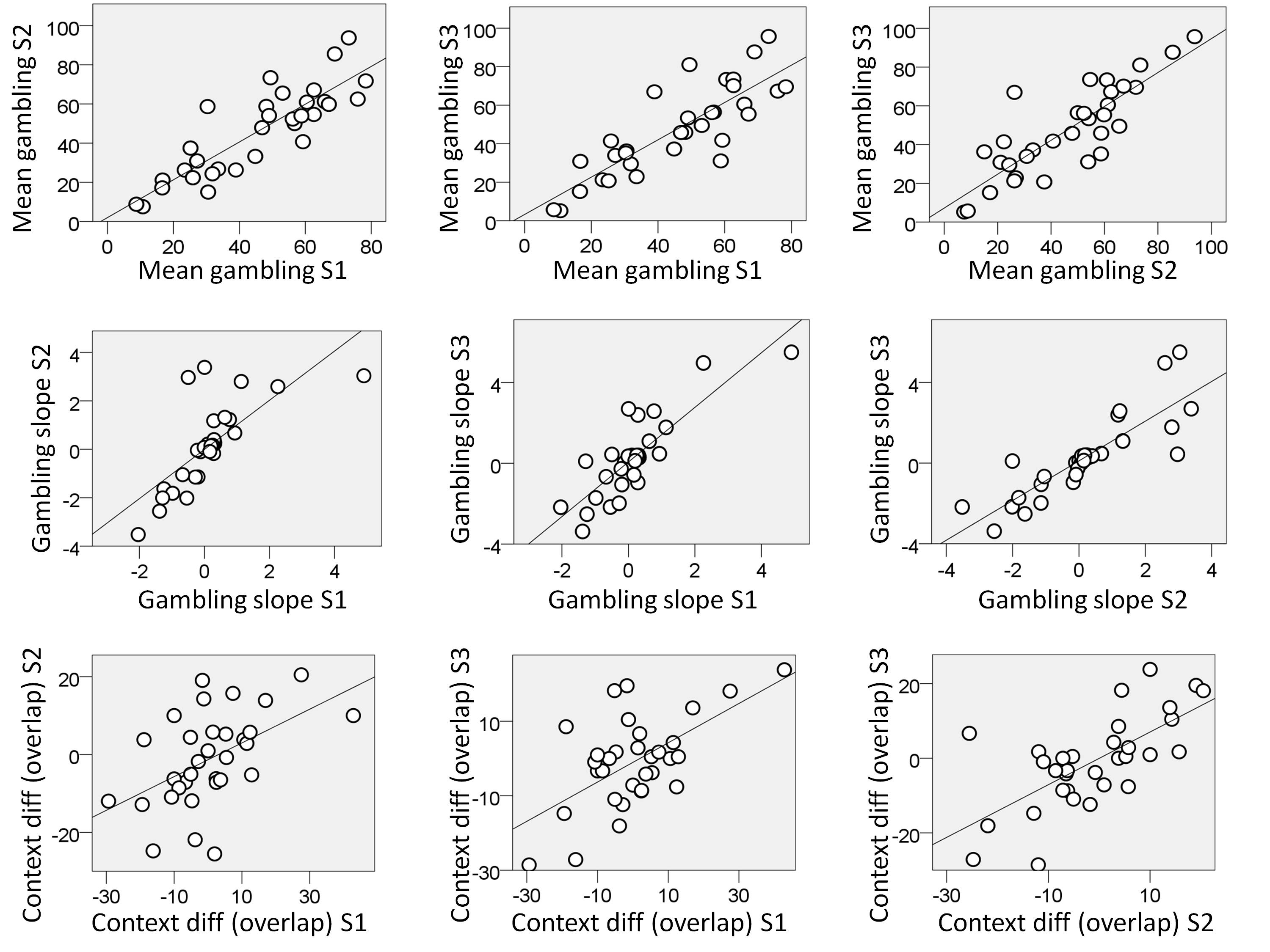


**Figure S2.** Relationship among equivalent indexes across different sessions. Different rows correspond to (i) mean gambling percentage, (ii) gambling slope, the beta weight associated with EV in a logistic regression model of gambling choice - positive and negative gambling slopes indicate an increased gambling with larger and smaller EVs respectively, (iii) difference in gambling percentage for EVs that were present in both contexts (£2-£5 range for low minus high-value context; a positive difference indicates greater gambling percentage in the low-value context). Different columns indicate sessions (S1 = session 1; S2 = session 2; S3 = session 3). All correlations are significant (p < 0.005).


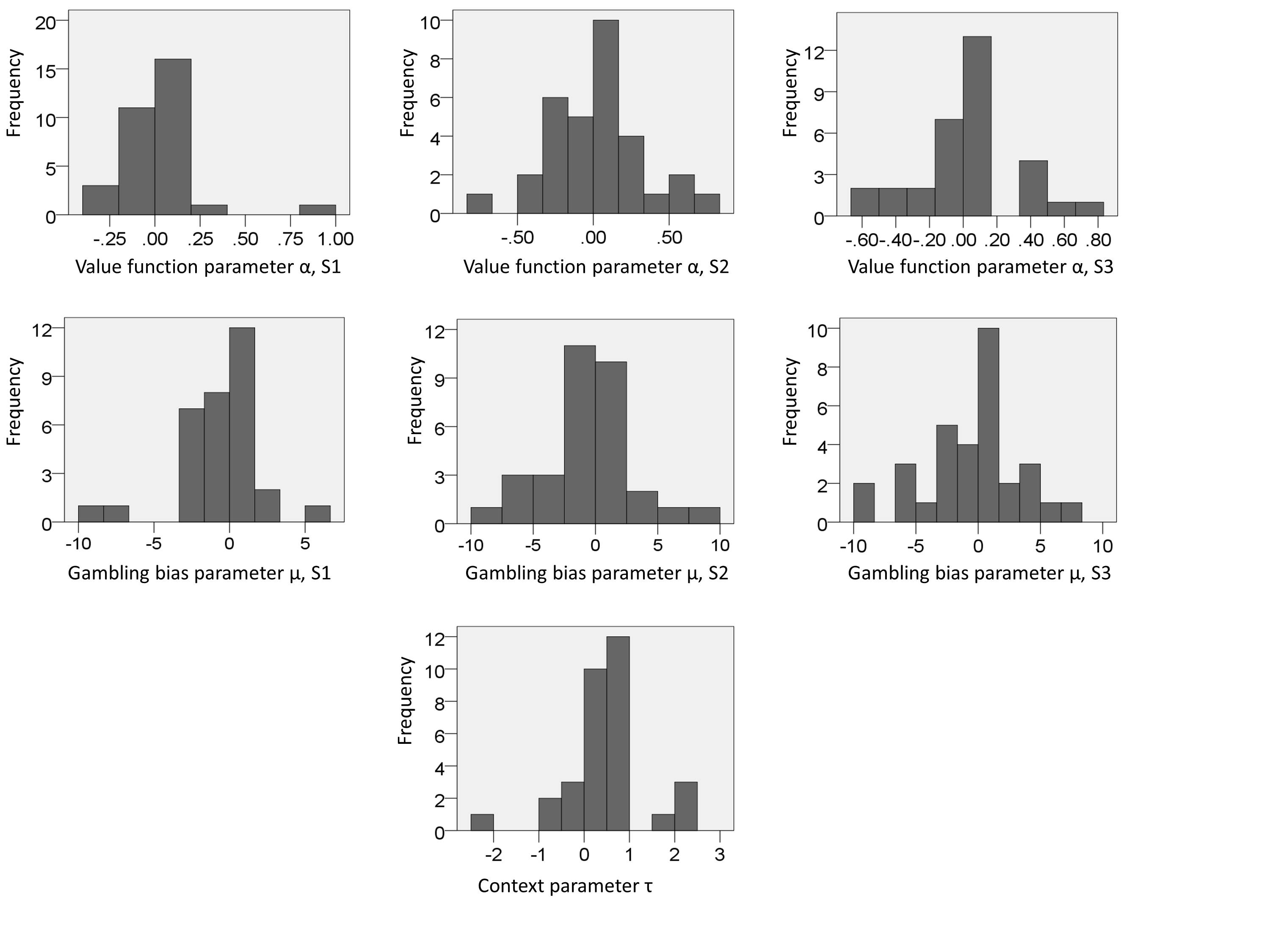


**Figure S3.** Distribution of the parameters across participants estimated from choice data using the best fitting model (according to model comparison performed using BIC scores). In this model, different value function parameters α and gambling bias parameters μ were estimated in each session, while a single context parameter τ was estimated across all sessions.

| Subjective State Questionnaire | Placebo Start | L-DOPA Start | P-value | Placebo End | L-DOPA End | P-value |
| --- | --- | --- | --- | --- | --- | --- |
| Alert 0 – Drowsy 10 | 0.22 | 0.15 | 0.88 | 0.59 | 0.78 | 0.80 |
| Calm 0 – Excited 10 | 0.81 | -0.70 | 0.01 | 0.74 | -0.85 | 0.02 |
| Strong 0 – Feeble 10 | 0.07 | 0.19 | 0.80 | 0.56 | 0.85 | 0.56 |
| Muzzy 0 – Clear-Headed 10 | -0.19 | 0.00 | 0.67 | -0.67 | -0.78 | 0.84 |
| Well-Coordinated 0 – Clumsy 10 | 0.11 | 0.07 | 0.93 | 0.19 | 0.37 | 0.65 |
| Lethargic 0 – Energetic 10 | -0.11 | -0.89 | 0.18 | -0.63 | -1.30 | 0.28 |
| Contented 0 – Discontented 10 | -0.22 | 0.30 | 0.07 | 0.41 | 0.37 | 0.92 |
| Troubled 0 – Tranquil 10 | 0.00 | 0.22 | 0.39 | -0.37 | -0.26 | 0.71 |
| Slow 0 – Quick Witted 10 | -0.04 | -0.70 | 0.15 | -0.04 | -0.81 | 0.15 |
| Tense 0 – Relaxed 10 | -0.33 | 0.26 | 0.17 | -0.67 | 0.30 | 0.05 |
| Attentive 0 – Dreamy 10 | 0.11 | 0.70 | 0.27 | 0.78 | 1.44 | 0.33 |
| Incompetent 0 – Proficient 10 | -0.22 | -0.33 | 0.82 | -0.37 | -0.74 | 0.47 |
| Happy 0 – Sad 10 | 0.07 | 0.15 | 0.82 | 0.30 | 0.19 | 0.75 |
| Antagonistic 0 – Friendly 10 | -0.26 | -0.52 | 0.39 | -0.63 | -0.41 | 0.26 |
| Interested 0 – Bored 10 | 0.37 | 0.78 | 0.32 | 1.33 | 1.74 | 0.27 |
| Withdrawn 0 – Sociable 10 | -0.44 | -0.26 | 0.51 | -0.74 | -0.56 | 0.65 |

**Table S1.** Scores represent differences in ratings in a subjective state questionnaire between baseline (i.e., before placebo or L-DOPA administration) and the start of the task or the end of the task. Questions were answered by marking a point on a line and responses were converted to a 0-10 scale. P-values shown are relative to corresponding paired t-tests and are not corrected for multiple comparisons. One variable (Calm-Excited) showed a p < 0.05 in both differences, however neither of the differences did survive Bonferroni correction for multiple comparisons.
